# A Chronic Complaint

**Published:** 1883-07

**Authors:** 


### A CHRONIC COMPLAINT.

Dr. Grohnwald's reflector has been accepted favorably in America, but instead of giving due honor to the inventor, they have "improved" it by adding a semi-circular piece of tin as a shade in front of the lens, and now the reflector is called an "American invention." Perhaps we will soon have this latest novelty imported into Germany, and it would not be a surprise to see it extensively introduced at an increased cost, because otherwise our Germans would not buy it. And this reminds us of many things which Germans only purchase after they have traveled through France, England or America. For instance, nail-files and scroll saws are shipped to England, returned at double the price and thus sold. Another anomoly of this kind is the mouth mirror manufactured by Messrs. Gessell Bros., of Berlin. These instruments with an ebony handle are thrown upon the market at m. 1.50 (38 cts.) The firm sends them to Philadelphia in lots of 1,000 at m. 1 (25 cts.) a piece; the dental depots of that city sell them to English firms, and from these again they are sold to German depots at m. 7.50 ($1.87) a piece. *Semper aliquid haeret.—Zahntechnische Reform.*

The honors are easy as between the old world and the new. We could instance many articles, the product of American genius, which are sold in Europe as of home invention. Snow's Saliva Ejector was not only stolen bodily, but insult was added to injury by copying the very illustrations and recommendations first used here, and thus were deceived the very elect, for the American Editor of Coleman's Surgery and Pathology borrowed the cuts and paraded it as a foreign invention.

For years the silvered and ground glass for the more popular styles of mouth mirrors were imported into this country by American manufacturers, who then mounted them and supplied handles in different styles, exporting them again to Europe, at, of course, an increased price. No mounted mirrors of this style were ever imported here, those of entire German manufacture selling in this county at about five dollars apiece.—[EDITOR.

